# Mobile Health Technology for Personalized Tobacco Cessation Support in Laos (Project Support Laos): Protocol for a Randomized Controlled Trial

**DOI:** 10.2196/89916

**Published:** 2026-05-21

**Authors:** Thanh Cong Bui, Phonepadith Xangsayarath, Phayvanh Keopraseuth, Khatthanaphone Phandouangsy, Khamsing Keothongkou, Vangnakhone Dittaphong, Cate Moriasi, Khue-Tu T Doan, Shweta Kulkarni, Chanthavy Soulaphy, Dalouny Xayavong, Viengphone Sangxayalath, Khanittha Sengdara, Michael S Businelle, Summer G Frank-Pearce, Jennifer I Vidrine, Damon J Vidrine

**Affiliations:** 1 Department of Family and Preventive Medicine, College of Medicine, University of Oklahoma Health Campus, Oklahoma City, OK, United States; 2 TSET Health Promotion Research Center, Stephenson Cancer Center, University of Oklahoma Health Campus, 655 Research Parkway, Suite 400, Oklahoma City, OK, 73104, United States, 1 7134746040; 3 Department of Communicable Disease Control, Ministry of Health of Lao People's Democratic Republic, Vientiane Capital, Lao People's Democratic Republic; 4 Ministry of Health of Lao People's Democratic Republic, Vientiane Capital, Lao People's Democratic Republic; 5 Secretariat of the National Tobacco Control Committee, Ministry of Health of Lao People's Democratic Republic, Vientiane Capital, Lao People's Democratic Republic; 6 Champasak Hospital, Champasak Province, Lao People's Democratic Republic; 7 Setthathirath Hospital, Vientiane Capital, Lao People's Democratic Republic; 8 The Matilda Centre, The University of Sydney, Sydney, Australia; 9 Department of Biostatistics and Epidemiology, Hudson College of Public Health, University of Oklahoma Health Campus, Oklahoma City, OK, United States; 10 Department of Health Outcomes and Behavior, Moffitt Cancer CenterTampa, FL, United States

**Keywords:** tobacco treatment, mobile health, Lao People's Democratic Republic, smoking cessation, mHealth-based intervention

## Abstract

**Background:**

Tobacco use remains the leading cause of preventable morbidity and mortality in Lao People’s Democratic Republic (Lao PDR). Despite the prevalence of cigarette smoking in Lao PDR (51% in men and 7% in women), no national tobacco treatment programs are available. Therefore, the development and evaluation of sustainable tobacco cessation interventions suitable for widespread adoption in Lao PDR are pressing public health needs.

**Objective:**

This project aims to adapt our theoretically and empirically based mobile health (mHealth) technology to help people quit smoking cigarettes in Lao PDR.

**Methods:**

Our mHealth approach includes a fully automated, interactive, personalized, smartphone-delivered intervention for behavioral treatment. This project includes 2 main phases. In the first phase, we use formative research methods to adapt our intervention content to the sociocultural context, language, and communication styles of Laotians. In the second phase, we conduct a randomized controlled trial to evaluate the efficacy of our mHealth intervention. In the trial, adult smokers are recruited through 2 national hospitals: Setthathirath Hospital in Vientiane and Champasak Hospital in Champasak Province. Participants (n=500) are randomized to either the standard care (SC; n=250) or automated treatment (AT; n=250) group. SC consists of brief advice to quit smoking delivered by research staff, self-help written materials, and a 2-week supply of nicotine replacement therapy (transdermal patches). AT consists of all SC components plus a fully automated smartphone–based treatment program that involves interactive and personalized proactive messages, images, or videos. The primary health outcome of the trial is biochemically confirmed self-reported 7-day point prevalence abstinence 12 months post study enrollment. Secondary outcomes include abstinence at 3 and 6 months post enrollment.

**Results:**

This study was approved by the ethical review boards of the respective domestic and international institutions. Data collection for the formative phase occurred from January 2022 to May 2023, and data analyses are ongoing. Data collection for the trial phase is ongoing and is expected to be completed by the end of 2026.

**Conclusions:**

If the proposed project is successful, it has the potential to transform health care services for tobacco treatment throughout Lao PDR and, ultimately, to significantly reduce tobacco-induced morbidity and mortality in the country. The AT’s potential for widespread adoption and sustainability is enhanced by the direct involvement of Lao governmental stakeholders at multiple national institutes. Furthermore, the US-Lao collaborative work and capacity-building activities in this project will contribute to creating a knowledge base for mHealth research applications and advancing mHealth research capacity in Lao PDR.

## Introduction

Tobacco use is the leading preventable cause of cancer death worldwide [1]. Tobacco also causes a disproportionately higher number of deaths in low- and middle-income countries (LMICs) [1,2]. Unfortunately, cessation treatments in LMICs are often unavailable or unaffordable for most people [1]. In Lao People’s Democratic Republic (Lao PDR), a recent national survey indicated that 51% of adult men and 7% of adult women smoke tobacco [3]. The Lao National Tobacco Control Committee (NTCC) has implemented several tobacco control efforts, such as taxing tobacco products, expanding smoke-free environments, requiring health warnings on cigarette packaging, and comprehensive bans on tobacco advertising. However, no national tobacco treatment programs are available [4,5]. Thus, the development and evaluation of sustainable tobacco cessation interventions suitable for widespread adoption in nations such as Lao PDR are pressing public health needs. Despite this need, no reports of efficacious tobacco cessation interventions in Lao PDR have been published.

To address this need, we propose a project that adapts our theoretically and empirically based mobile health (mHealth) technology to help people in Lao PDR quit smoking cigarettes. This mHealth approach includes a fully automated, interactive, personalized, smartphone-delivered intervention for behavioral treatment, delivered through our Insight platform. The World Health Organization (WHO) acknowledges the potential of mHealth interventions to transform the face of health service delivery across the globe, including in least developed countries [6]. The data from the International Telecommunication Union, which is the United Nations’ official source for global information technology statistics, show that mobile cellular subscriptions now make up >98% of voice subscriptions in least developed countries [7]. The data also showed that Lao PDR was in the top 5 countries globally for increased mobile broadband subscriptions from 2016 to 2017 [7], suggesting substantial growth in mobile broadband coverage and usage in the near future. Most smartphones (80%) in Lao PDR are Android-based [8] and can function in Lao script. In summary, smartphone ownership is clearly increasing in Lao PDR, providing an ideal yet largely untapped mechanism to deliver smoking cessation treatment.

This protocol paper describes our proposed project that aims to adapt our theoretically and empirically based mHealth technology to help Lao people quit smoking cigarettes. The project is funded by a phased R21/R33 grant (R33CA253600) from the US National Cancer Institute (NCI). The project includes 2 main phases. In the first phase (ie, the R21 phase), we use formative research methods to adapt our intervention content to the sociocultural context, language, and communication styles of Laotians (aim 1). In the subsequent R33 phase (aim 2), we conduct a randomized controlled trial (RCT) to evaluate the efficacy of our mHealth-based intervention. The primary health outcome of the trial is biochemically confirmed self-reported 7-day point prevalence abstinence at 12 months post study enrollment. Secondary outcomes include abstinence at 3 and 6 months post enrollment. This project also supports mHealth research capacity building in Lao PDR and sustaining the US-Lao PDR research network by supporting Lao investigators through in-person workshops, in-service trainings, online trainings, manuscript preparation, and future mHealth research collaboration and grant applications (aim 3). The project has the potential to transform health care services for tobacco treatment throughout the country and, ultimately, to significantly reduce tobacco-induced morbidity and mortality.

## Methods

### Ethical Considerations

This study was approved by the Lao National Ethics Committee for Health Research (NECHR #50) and by the institutional review board (IRB) of the University of Oklahoma Health Campus (IRB#: 12189), which was selected to serve as the single IRB of record for participating US domestic institutions. Our collaborative hospitals, Setthathirath Hospital (SH) and Champasak Hospital (CH), rely on the Lao National Ethics Committee for Health Research IRB. All potential participants undergo an informed consent process conducted in the Lao language by local research staff. Written consent is obtained from all who choose to participate. Participant privacy and confidentiality is carefully maintained. The currently approved IRB protocol version is dated January 20, 2022. Participants were compensated an amount in Laotian kip equivalent to US $15 after completing each in-clinic visit at baseline and at 3-, 6-, and 12-month follow-ups.

### Overall Study Design

This study uses a mixed methods research design with 2 main phases. The R21 phase includes qualitative and pilot RCT components to adapt and validate our intervention content to the sociocultural context, language, and communication styles of Laotians. In the R33 phase, we conduct a 2-group single-blind RCT to compare the efficacy of 2 smoking cessation interventions: the mHealth-based automated treatment (AT; n=250) versus standard care (SC; n=250). We utilized the CONSORT (Consolidated Standards of Reporting Trials) [9] and SPIRIT (Standard Protocol Items: Recommendations for Interventional Trials) [10] checklists in the preparation of this paper.

### Conceptual Framework for AT

Our AT intervention is based on the phase-based model (PBM), a theoretical framework that is specific to smoking cessation [11,12]. PBM partitions the cessation process into 4 phases: motivation, preparation (precessation), cessation (quit date to 4 weeks post quit), and maintenance (up to 6 months post quit); the AT focuses on the latter 3 phases. PBM helps identify challenges and opportunities that smokers face at each phase, explains underlying phase-specific mechanisms, and facilitates the selection of intervention components and measures. Using data from smartphone-delivered weekly assessments (see more details in the *Intervention* Conditions and *Measures* and Assessment Strategy sections), the AT will dynamically target several putative mechanisms, mainly those that are relevant across phases and have been reliably associated with long-term abstinence in previous studies: withdrawal and craving, motivation to quit, positive and negative affect, coping with stress and urges, and self-efficacy [11,13-16]. Specifically, AT will promote skills to reduce and cope with withdrawal and craving. Motivation plays a critical role in initiating and successfully maintaining change [17-20]. Because motivation is dynamic, AT is well suited to target shifts in motivation to quit. Stress and negative or positive affect mechanisms are important targets in the preparation and cessation phases [11,13] due to their strong dose-response relationship with smoking-related acute events [21]. They have been associated with relapse and poor treatment outcomes in numerous studies [22-25]. Self-efficacy, in the context of smoking, is reflected in one’s confidence to not smoke in different challenging situations [26] and is among the best predictors of smoking treatment outcomes [15,23,27-29] and relapse [30]. Therefore, the AT will target these key mechanisms.

### Study Sites and Setting

Two hospitals, SH in Vientiane Capital and CH in Champasak province, serve as the main recruitment sites for this study. Vientiane Capital is in the central area and the most populous city in Lao PDR, while Champasak is the most southwestern province and borders Thailand and Cambodia. SH and CH are public general hospitals under the Lao Ministry of Health’s (MOH) management. In 2018, the MOH and Japan International Cooperation Agency selected SH and CH to receive grant aid of approximately 1.7 million USD from the Government of Japan to increase their capacities in providing high-quality medical care and services [31]. The grant supports improved facilities (eg, construction of new buildings or rooms) and equipment (eg, computed tomography scan and computers) to help the hospitals serve more patients in the region. The goal of this capacity enhancement is to achieve universal health coverage, defined as coverage such that all people receive appropriate health promotion, and preventive, curative, and rehabilitative health services at affordable costs [31]. SH and CH are also among the major implementation sites for several national disease control programs, including HIV, tuberculosis, and severe acute respiratory syndrome. These missions position the 2 hospitals as ideal venues to offer smoking cessation treatment, and the improved facilities and resources afford a solid foundation for AT adoption and implementation.

Each year, SH serves approximately 20,000 inpatients and 90,000 outpatients from all over Lao PDR, mostly from the north and central regions. CH is the largest and the top-referral hospital in the southern region. CH serves patients from all nearby provinces, of which the total population is approximately 1.5 million people.

### Phase 1 (R21): Adaptation of the AT Intervention Content

#### Overview

**Figure 1.**
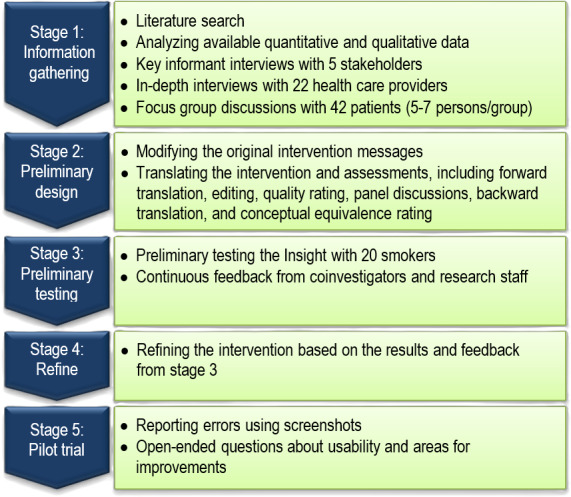
Adaptation of the automated treatment intervention content.

Because we adapted an existing theoretically based AT intervention from our previous works in the United States [32-34] and in Cambodia [35,36], we used the cultural adaptation of evidence-based interventions approach [37,38]. Although several models have been proposed to guide cultural adaptation, there is considerable consensus that cultural tailoring can be organized into 5 stages: information gathering, preliminary design, preliminary testing, refinement, and final trial [38]. Figure 1 summarizes our R21 research activities.

#### Stage 1: Information Gathering

##### Literature Searches and Key Informant Interviews

We conducted literature searches, analyzed quantitative results of the Lao National Adult Tobacco Survey [3,39], and discussed our intervention approach with 5 key stakeholders (at the MOH, NTCC, and University of Health Sciences in Lao PDR) to determine how well our original intervention would fit the needs and preferences of the target population. Information gathered from these activities helped us to shape the proposed project. For example, National Adult Tobacco Survey data showed that ~90% of male tobacco users smoke cigarettes [3]; thus, we adapted the AT intervention to target cigarette smokers first. MOH and NTCC stakeholders confirmed that the AT has great potential regarding scalability and sustainability. The NTCC’s previously developed materials in Lao for smoking cessation support were used to modify our intervention content library.

##### In-Depth Interviews

We conducted in-depth interviews with 22 health care providers at participating hospitals to learn more about how they typically motivate patients to quit smoking and stay abstinent. They could develop intervention messages if they wish or pinpoint the sources of treatment content that they use. We used purposive sampling to select a diverse sample regarding age and specialty.

##### Focus Group Discussions

We also conducted 7 focus group discussions with 42 patients who smoked (5‐7 persons per group) to identify additional factors that motivate patients to quit smoking and stay abstinent, barriers to quitting, and strategies to overcome these barriers. We used purposive sampling to select a diverse sample with regard to age, residence (urban vs rural), disease type, and cessation phase per the PBM. We conducted separate group discussions for men and women.

##### Participant Eligibility for the Qualitative Components in the R21 Phase

Inclusion criteria for all health care providers in the R21 phase included (1) age ≥18 years and (2) the ability to provide written informed consent to participate. Inclusion criteria for all patient participants who smoked in stages 1 and 3 of the R21 phase were (1) age ≥18 years, (2) self-reporting current combustible cigarette smoking (smoked ≥100 cigarettes in lifetime and currently smoke ≥1 cigarette per day), (3) planning to quit in the next 6 months or having actually made a quit attempt, and (4) the ability to provide written informed consent to participate.

##### Interview Procedure and Qualitative Data Analysis

For all qualitative components in the R21 phase, we used interview guides with open-ended questions. All interviews and discussions were recorded using a digital voice-recording app on 2 encrypted smartphones and were transcribed verbatim. Two senior research coordinators independently coded the first 2 transcripts in English. The US principal investigator (PI), Lao Site PI, and a Lao co-investigator at NTCC met with the 2 senior research coordinators to review the coded transcripts and to discuss and resolve discrepancies. Then, the 2 research coordinators continued to code other separate sets of transcripts. Each subsequent coded transcript was reviewed by at least 1 PI or co-investigator. Qualitative data were analyzed using thematic content analysis with the aid of the R-based Qualitative Data Analysis software package [40]. Themes were based on the purpose of each component and on theoretical constructs of the PBM.

### Stage 2: Preliminary Design

#### Overview

We integrated information gathered in the first stage to modify the original intervention. For example, using literature search and qualitative findings, we expanded our treatment content library to include strategies for coping with craving or urges that are commonly used in Southeast Asian culture (eg, chewing on ginger or lemon, chatting with friends via apps, or playing e-games on phones to keep hands busy). The original English treatment content library and the NTCC’s available materials are mainly text based with some images.

#### Translation of Study Materials

We translated all English intervention materials and assessments for use in the R33 phase into Lao, mirroring the WHO’s recommended methodology [41,42]. First, a health scientist, whose native language is Lao, led the forward translation process, focusing on conceptual (vs literal) meaning and comprehensible language for the broadest audience. Second, a more senior bilingual health expert (the Lao Site PI or a Lao co-investigator) reviewed the translation, discussed disagreements with the forward translator, edited, and finalized the forward translation. Third, 2 other bilingual health professionals independently rated the quality of the translation on 5 dimensions: conceptual equivalence, clarity in meaning, comprehensibility, use of common simple language, and cultural appropriateness. Any discrepancies were discussed to reach consensus on translation or editing. Fourth, we conducted 6 panel discussions (1 with female smokers, 1 with male smokers, and 1 with mixed-sex health care professionals at each participating hospital; 5‐7 panelists per group). The aim of this step was to evaluate material comprehensibility as well as linguistic and cultural appropriateness for the target populations, particularly to identify linguistic differences by regions (north vs south) in Lao PDR and to find and use common words. Fifth, 2 other bilingual health professionals independently backward translated the materials into English. Finally, the PI and Lao Site-PI independently reviewed and rated the backward translations for conceptual equivalence with the original English versions.

### Stages 3 and 4: Preliminary Testing and Refinement

We loaded the intervention content onto the Insight platform and assessments into REDCap (Research Electronic Data Capture) and preliminary tested them (on smartphones or tablets) with research team members and some people who smoked. We used their feedback to refine the intervention and assessments. Additionally, we systematically documented continuous feedback from Lao investigators and staff members who implemented stages 2 and 3 for intervention adaptation and refinement. Critical changes at this stage (eg, major deviations from the original intervention) were made in consultation with the PIs.

### Stage 5: Pilot Trial

We conducted a 3-month pilot efficacy trial with 50 participants who smoked. Activities at this stage were designed to mirror the full efficacy trial in the R33 phase, except that only 50 participants were recruited and the smoking treatment lasted 3 months. In addition to the efficacy outcomes, we collected data for further adaptation and refinement. Specifically, at the baseline, we instructed participants to take screenshots of the phones and asked them to do so whenever they saw a message that they did not understand, that had an error, or that needed revision. We asked participants to send us these screenshots together with descriptions of issues via Insight or emails. At the follow-up, we checked for and discussed all stored screenshots with participants to elicit their suggestions for revision, and we asked AT participants open-ended questions regarding usability and areas for improvement in the AT.

### Phase 2 (R33): Conduct a Single-Blind, 2-Group RCT to Evaluate the Efficacy of AT

#### Overview

Participants (n=500) are patients who smoke recruited through SH in Vientiane and CH in Champasak province in Lao PDR. Consenting participants are randomized to one of the 2 study groups: AT (n=250) or SC (n=250). SC consists of brief advice to quit smoking delivered by research staff, self-help written materials, and a 2-week supply of nicotine replacement therapy (NRT) in the form of transdermal patches. AT consists of all SC components plus the fully automated smartphone–based treatment program that involves interactive and tailored proactive messages and images for 6 months. Participants complete in-clinic assessments at baseline and at 3, 6, and 12 months following study enrollment. Participants also complete brief weekly assessments via smartphone during the AT period so that the intervention content can be tailored to their PBM phases and constructs. Participants are blinded to their group assignment. The primary health outcome of the RCT is biochemically confirmed self-reported 7-day point prevalence abstinence 12 months post study enrollment. Secondary outcomes include abstinence at 3 and 6 months post enrollment.

#### Design Considerations

Several aspects of the project, as described below, were carefully considered prior to deciding on the proposed design.

##### Provision of NRT

We considered a 2×2 fully crossed randomized design to differentiate the effects of pharmacological support (NRT) versus behavioral treatment (AT). However, NRT helps address nicotine withdrawal and craving [11,43] and increases quit rates, and the US Public Health Service Guidelines state that NRT should be considered the minimal standard of care [44]. Thus, it would be unethical to have a study group without NRT in a large trial. On the other hand, providing too much NRT may mask the effect of AT and affect the sustainability of the program. Most quitlines in the United States provide a minimum of a free 2-week starter kit of NRT, and we followed this practice.

##### Provision of Smartphones

We considered using a more real-world approach, in which only individuals who already owned smartphones would be eligible for study entry. However, we decided that loaning smartphones to those who either do not own or own an incompatible smartphone would actually provide a more thorough and realistic estimate of AT’s potential. This is especially true given the trend in Lao smartphone ownership over the past few years, which indicates that ownership will be nearly ubiquitous in the near future. In our Cambodian pilot study, 88% (n=50) returned the project phones after 2 months.

### Participants Recruitment and Eligibility

#### Overview

All nonemergency patients coming to SH and CH first go to a reception desk to receive a queue number and a basic medical form. A flyer that introduces this study is attached to the basic medical form. Additionally, we proactively recruit patients at the respiratory disease screening units (RDSUs) and departments or clinics specializing in women’s health (eg, gynecology, and breast and gynecologic cancer). Due to the national organization and implementation of disease control programs, all patients visiting SH or CH with respiratory symptoms are first examined at the RDSUs for type of disease, severity, and whether the disease is included in a national disease control program (eg, tuberculosis or severe acute respiratory syndrome). Patients are then referred to appropriate departments and clinics or disease control units. The RDSUs at SH and CH see about 40 and 20 patients daily, respectively. Of these, 70% are men; 60% to 70% of male patients smoke cigarettes (no estimate available for women; Setthathirath Hospital, unpublished data, 2018 and Champasak Hospital, unpublished data, 2018). Research staff proactively screen as many patients visiting the RDSU as possible for eligibility and invite eligible individuals to participate. Research staff also proactively screen as many women visiting the women’s health clinics as possible to enroll female smokers.

In our Cambodian pilot study at a clinic with fewer patients, 86% (51/59) of the identified eligible smokers consented to participate, and we recruited 2‐5 participants per day; we expect a similarly high consent rate for this study. Together, SH and CH see >160,000 unique patients each year. Based on the smoking prevalence in the general population (51% in men and 7% in women), we estimate that approximately 40,000 male patients and 5600 female patients at SH and CH are current smokers. Assuming, conservatively, that only half of these smokers are eligible for the study, and even if only half of eligible smokers want to participate, there will be 10,000 available male and 1400 available female smokers in a 1-year recruitment period. Thus, our goal to enroll 500 participants (<5% of eligible smokers) at SH and CH is highly feasible.

#### Inclusion Criteria

Trial inclusion criteria include patients who are (1) aged ≥18 years, (2) self-reporting current combustible cigarette smokers (smoked at least 100 cigarettes in lifetime and currently smoke ≥1 cigarette per day), (3) willing to set a quit date within 2 weeks of study enrollment, (4) able to provide written informed consent to participate, and (5) able to read Lao (score ≥4 points on the Rapid Estimate of Adult Literacy in Medicine–Short Form [45]).

#### Exclusion Criteria

Exclusion criteria include (1) history of a medical condition that precludes the use of NRT, (2) ineligibility to participate based on medical or psychiatric conditions diagnosed by a physician or clinician, and (3) enrollment in another cessation program or current use of other cessation medications.

### Baseline Assessment and Randomization

Enrolled participants complete a 45-minute tablet-delivered baseline assessment, managed and delivered by REDCap [46,47]. The use of REDCap enhances both accurate data collection across sites and timely and secure data transmission. Research staff assist participants in completing the assessment if needed. Participants are assigned to a treatment group using simple randomization (1:1) and were blinded to their study group. All participants complete a brief training session on smartphone use and the Insight app. Smartphones are loaned to participants who need them.

### Intervention Conditions

#### Standard Care

Participants randomized to SC receive brief advice to quit smoking delivered by research staff, NTCC’s self-help materials (developed based on the WHO’s “A guide for tobacco users to quit” [48]), and a 2-week supply of NRT (patches). SC participants are asked to complete weekly 4-item smartphone-delivered assessments about their diet (see explanation in the *Brief Weekly Smartphone Assessments* section) for a 6-month period.

#### Automated Treatment

Participants in the AT group receive the SC components (except the dietary assessments) plus proactive personalized messages for smoking cessation. The AT content is adapted from the team’s previous efforts, is informed by the R21 phase outcomes, and is designed to tap the theoretical mechanisms described in the PBM. That is, as we described in a prior study [35], AT content is designed to increase motivation, self-efficacy, and use of coping skills, while reducing nicotine withdrawal symptoms and stress. AT begins immediately after enrollment and continues for a 26-week period (about 2 messages per day). The AT approach allows for several levels of personalization for each participant. First, at baseline, participants are asked several questions about their biological sex, past quit attempts, and the presence or fear of specific health conditions. Treatment content tailored to these responses is automatically delivered throughout the treatment period. Second, there are different bins of treatment content for different cessation phases to ensure that AT targets critical mechanisms of each participant’s PBM phase, which may dynamically change during treatment. Third, participants are asked to complete brief (4 items) smartphone-delivered assessments via the Insight app during each week of the AT course. These questions vary depending on each participant’s phase (eg, current level of intrinsic motivation for preparation or maintenance phases or smoking status in the past week and self-efficacy level for all phases). Treatment content (eg, types and frequencies of messages) for the following week is based on responses to these weekly assessments and participant phases (eg, level of self-efficacy or past-week smoking status).

**Figure 2.**
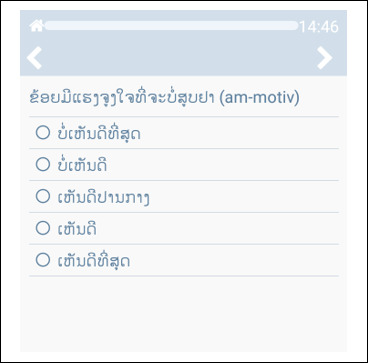
Screenshot of the Insight app.

Our Insight platform [49], which includes a web-based central management system and an app, is used to manage and deliver the AT and weekly assessments. The Insight app will automatically deliver all interventions and assessments as prescheduled, with or without an active cellular network connection, thus ensuring timely and reliable AT delivery throughout the 6-month treatment period. Through 1-click buttons in the Insight app, participants can call or message project staff if they have questions or need assistance. Insight allows our AT to function autonomously and minimizes human involvement, making the approach very affordable for large-scale implementation in LMICs. Most importantly, Insight enables complex built-in algorithms and branching logic, allowing us to create and deliver dynamically and individually tailored treatment content as described above. Our pilot results in Cambodia demonstrate that Insight works very well in Southeast Asia; it is compatible with the GSM and 4G data networks, properly delivers messages and weekly assessments, and reliably collects and transfers data to our encrypted server. Our preliminary work also shows that the Insight platform and app work well with the Lao script (Figure 2).

### Measures and Assessment Strategy

#### In-Clinic Assessments at Baseline and at 3-, 6-, and 12-Month Follow-Ups

Three types of data are collected at each visit: self-report data (Table 1) collected by the Health Insurance Portability and Accountability Act–compliant REDCap program on a tablet, expired air carbon monoxide (CO) assessed with a CO monitor (Vitalograph BreathCO) by research staff to verify smoking status biochemically, and medical record data (to collect clinical information, such as current diagnosed health conditions for AT personalization). The REDCap assessment is administered at baseline (~45 minutes to complete) and at each follow-up in-clinic visit (~20 min). For measures that have not been validated in Lao, we adapted them as described in the R21 phase.

**Table 1.** Study assessments conducted in-clinic at baseline, 3-, 6-, and 12-month follow-ups, and weekly via the Insight app.

Variable type and measure	Baseline	Weekly^a^	In-clinic follow-up (3, 6, and 12mo)
Descriptors or potential moderators			
Demographics; health literacy [50]	X		
Health conditions and comorbidities; substance use [51,52]	X		X
Dependence—heaviness of smoking index [53]	X		
Adherence to treatment app			
Duration of phone on or off; numbers of messages or images or videos delivered, opened, and marked as viewed; numbers of weekly assessments opened and completed; data syncing frequencies (manual and automatic)	X^b^	X^b^	X^b^
Phase-based model mechanisms			
Wisconsin Smoking Withdrawal Scale [54]	X		X
Reasons for quitting (intrinsic, extrinsic) [20]	X		X
Contemplation ladder [17,18]	X	X^c^	X
Kessler Psychological Distress Scale (K10) [55]	X	X^c^	X
Positive and Negative Affect Schedule [56]	X		X
Self-efficacy (related to smoking cessation) [30]	X	X^c^	X
Primary outcome			
Smoking status (includes number of quit attempts, and days abstinent) [57]	X	X^c^	X
Expired carbon monoxide [57]	X		X

a Delivered via Insight app on smartphone.

b Documented by digital date and time stamps in the Insight’s activity log.

c Brief versions of the scales.

#### Brief Weekly Smartphone Assessments

Participants are asked to complete weekly assessments via smartphone for 6 months, delivered by the Insight app. Participants in the AT group receive 4‐7 smoking-related questions, depending on their cessation phase (Table 1). Although these treatment-driving questions can be considered part of the AT, we attempt to balance the effects of these weekly contacts between the treatment groups; SC participants are also asked to complete a weekly 4-item assessment via the Insight app with questions about diet. This consistency across participants also minimizes the chances of inadvertent revealing of participants’ assigned interventions. Responses are temporarily stored on smartphones and sync to our secure server whenever a connection is active; thus, we have near real-time access to the data. To carefully track participants’ completion of weekly assessments and adherence, data plans are provided for 6 months for both groups.

#### Primary Outcome

The primary outcome is smoking status at 12 months post enrollment. Abstinence is defined as biochemically confirmed self-reported 7-day point prevalence abstinence with expired CO <6 ppm [57]. Secondary outcomes include 3- and 6-month abstinence. We will consider several other common outcomes, such as continuous and sustained abstinence, number of quit attempts, and length of abstinence.

### Participant Tracking and Retention Procedures

We use various approaches to maximize our follow-up rate. These include (1) reminders via phone calls, messages through the Insight home screen, and Insight push notifications before the follow-up visit; (2) offering follow-up assessments on different days and times to accommodate schedules; (3) collecting other phone numbers and home addresses of participants; (4) obtaining the names and phone numbers of at least 3 collaterals (eg, relatives and friends) who can help locate the participant should the participant move or otherwise lose contact during the study; and (5) financially compensating participants for the time and costs associated with study participation (eg, transportation, child care).

### Data Analysis Plan

#### Overview

The study’s hypothesis is, at the 12-month follow-up, 7-day point prevalence biochemically confirmed abstinence (primary outcome) will be higher in the AT (vs SC) group.

The primary abstinence analysis will be intention-to-treat 7-day point prevalence biochemically confirmed abstinence; patients not completing follow-up assessments will be considered smokers. However, we will explore other ways of dealing with missing data as described in the *Missing Data and Dropouts* section. To estimate the effect of AT on intention-to-treat 7-day point prevalence biochemically confirmed abstinence rates while accounting for the potential clustering of participants recruited from the 2 clinic sites, we will use generalized linear mixed models (GLMM) analysis as reported in our previous studies [35]. Intervention groups (AT vs SC) will be estimated as a fixed effect, while the clinic will be modeled as a random effect nested within treatment condition. Sex can also be modeled as a random effect nested within clinic and treatment condition. Specifically, unadjusted and adjusted log binomial mixed models will be used to estimate the relative risk and 95% CI of the primary outcome of intention-to-treat biochemically confirmed abstinence in the AT (vs SC) group. Although the groups should be similar in baseline characteristics due to randomization, we will explore models that control for any demographic or clinical variables that differ between treatment groups at baseline (eg, sex, nicotine dependence, and reading level). We will also use GLMMs to examine changes in abstinence rates over time, while accounting for relevant baseline covariates. Similar GLMM or linear mixed model methodology, as appropriate for each outcome variable, will be used to examine other smoking-related variables, such as continuous abstinence, prolonged abstinence, and quit attempts. This project focuses on the latter 3 PBM phases, and thus, we will explore phase-specific outcomes, such as abstinence attainment and the number of days smoking or abstinent.

Based on our previous work and assuming attenuation of abstinence rates across the follow-up visits, we anticipate that the 7-day point prevalence abstinence at the 12-month follow-up will be 8% in the SC group and 17% in the AT group. With 250 participants in each group and assuming a 2-sided *α* of .05, we will have 86% power to detect a difference of 9% in 12-month 7-day point prevalence abstinence in the overall sample. It is unknown whether factors associated with smoking cessation differ by sex; it seems likely given the variation in smoking prevalence between sexes. Thus, we chose a sample size that gives 80% power to detect this difference in a subgroup analysis of only males (n=425, 85% of the sample). The subsample of female participants (n=75) gives 80% power to detect a 25% difference in 12-month 7-day point prevalence abstinence.

Potential treatment mechanisms will be examined via mediation analyses, with the intervention group (AT vs SC) being the independent variable, abstinence at 12 months being the outcome variable, and the hypothesized mechanisms (motivation, self-efficacy, stress, and negative affect) being potential mediators. The PROCESS macro for SPSS or SAS [58,59] or an equivalent method will be used to identify variables (eg, motivation, self-efficacy) that mediate the relationship between treatment condition and smoking cessation outcomes. This method uses an ordinary least squares path analytic framework to estimate direct and indirect effects in single and multiple mediation models, and bootstrapping methods are incorporated to generate CIs.

#### Missing Data and Dropouts

Treating participants lost to follow-up as smoking is a widely used strategy in smoking cessation studies. However, some researchers point to problems with this approach, especially when comparing treatment arms with differential dropout rates [60]. We will conduct sensitivity analyses to test for treatment differences assuming different missing data mechanisms. For example, we will consider a multiple imputation approach based on smoking-related patient characteristics at baseline, as well as demographics to account for potential missing-at-random mechanisms. We will also explore pattern-mixture and selection models to account for potential (and likely) missing-not-at-random mechanisms [61]. Similar findings based on these analyses will strengthen our study conclusions.

### Capacity Building

Aim 3 is to advance mHealth research capacity in Lao PDR and sustain the US-Lao PDR research network by supporting Lao investigators through in-person workshops, in-service trainings, online trainings, manuscript preparation, and future mHealth research collaboration and grant applications.

The long-term expected outcomes of our capacity-building strategy are increasing Lao investigators’ proficiency in health sciences, nationwide dissemination of AT, and sustaining the US-Lao mHealth research network. By participating in all steps of the project, the Lao research team will gain proficiency in health science research, including mixed methods, theory-based intervention in tobacco cessation, and mHealth-based intervention and assessment. Proficiency in health sciences can be evaluated by joint publications, publications on which Lao researchers or trainees are the first author, and the number of future joint grant applications.

## Results

**Figure 3.**
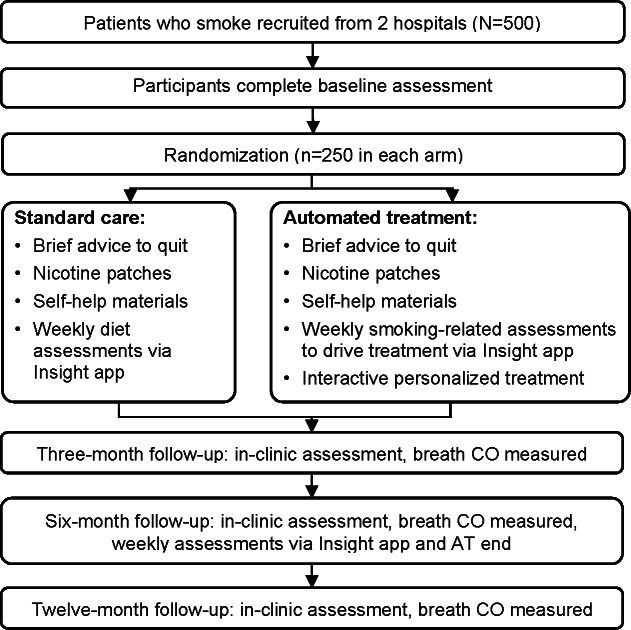
Trial schema. AT: automated treatment; CO: carbon monoxide.

**Figure 4.**
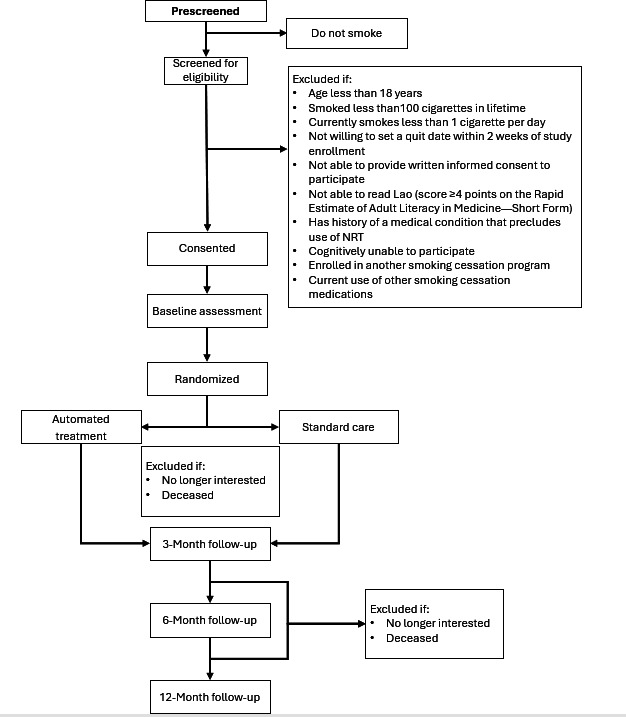
CONSORT (Consolidated Standards of Reporting Trials) flow diagram. NRT: nicotine replacement therapy.

The data collection for the formative R21 phase started in January 2022, following the necessary institutional approvals, and was completed in May 2023. A total of 69 individuals were recruited to participate in the qualitative components, including key informant interviews, in-depth interviews, and focus groups. Qualitative data analysis for the R21 phase contributed to the validation and adaptation of our intervention content to the sociocultural context, language, and communication styles of Laotians. After developing the intervention materials, we tested the intervention with 50 patients in a pilot RCT. The pilot trial data have been collected, cleaned, and analyzed. There was no loss to follow-up at the end of 3 months. The results indicated high feasibility of the intervention: 90.7% (of more than 300) study messages, events, and weekly assessments were delivered, and 88.7% of these were read or completed [62]. AT was also highly acceptable (eg, all AT participants agreed or strongly agreed that the message content was easy to understand). We presented the findings from the quantitative and qualitative components of the R21 phase at scientific conferences [62,63], and we are preparing manuscripts using these results, expected to be published in 2026. Participant enrollment for the main RCT (R33 phase) started in January 2024 and is still ongoing. The schematic representation of the RCT is shown in Figure 3, and the CONSORT flow diagram is shown in Figure 4. Data collection is expected to conclude by December 2026.

## Discussion

### Principal Findings

Cigarette smoking represents a major public health problem in LMICs including Lao PDR, but there is a lack of national smoking cessation treatment programs. Mobile phone–delivered text messaging has been identified as one of the most affordable interventions for smoking cessation [64] and has been endorsed and used by several international organizations, including the WHO and the US NCI, in their global tobacco control efforts [65,66]. There is also evidence that mHealth interventions are feasible in Lao PDR. For instance, the Lao MOH used SMS in a real-time reporting system for vaccine administration and monitoring [67,68]. The Lao MOH’s receptiveness and support of mHealth solutions increase the potential for sustainability and widespread adoption of our proposed mHealth intervention approach. In this study, we evaluate our automated mHealth intervention—AT—for smoking cessation in Lao PDR. Given AT’s potential as a feasible, scalable, and highly affordable stand-alone intervention, our approach is appropriate for use in LMICs and could substantially reduce smoking prevalence and smoking-related morbidities in the Lao population.

Several aspects of the proposed work are innovative. First, our AT is theoretically and empirically based. While several theory-based intervention studies (including our own) have been conducted in developed nations, efforts to adapt and disseminate these interventions to LMICs, such as Lao PDR, are lacking. In fact, there is no publication of theoretically based smoking cessation research in Lao PDR. Even in the United States, where numerous smartphone apps for smoking cessation are available, only 4% of the top 50 apps in leading app stores have any scientific basis [69]. Our proposed project is the first effort in Lao PDR to adapt an mHealth intervention for smoking cessation that is theoretically and empirically based and culturally tailored. Second, this project is a close collaboration between US investigators and Lao stakeholders at many institutions, including national and regional hospitals, the Lao MOH, and the NTCC. The US and Lao researchers are committed to a long-term partnership to build and sustain a national mHealth treatment program for tobacco cessation in Lao PDR. This committed partnership will provide critical infrastructure to support the future widespread adoption and sustainability of AT in Lao PDR. Finally, through collaboration and capacity-building activities, this project will be the first to train Lao investigators in mHealth research methodology and will strengthen their overall competencies in this field. Altogether, the project will lay the groundwork for a sustained US-Lao mHealth research network that will support future high-quality mHealth research projects in Lao PDR.

This study has some limitations. The use of Insight and smartphones may currently exclude some very low-income smokers. However, the data show that smartphone ownership is nearing ubiquity globally, including in Lao PDR. Thus, our proposal to use Insight and smartphones for AT delivery represents an attempt to utilize powerful technology while maximizing reach and impact. This study may have limited power to detect a difference in the primary outcome in the female subsample of the RCT. We acknowledge the importance of ensuring that our mHealth-based AT program works well for women. Thus, in the R21 phase, we recruited equivalent numbers of male and female smokers for qualitative interviews or discussions. However, given the limited timeframe and budget of the R33 phase (ie, the full-scale RCT), we must guarantee a sufficient sample size to demonstrate the efficacy of AT in at least the majority group of smokers (ie, men) if subgroup analysis is needed. Thus, we cannot oversample female smokers, but we will ensure that female smokers are represented in a manner that aligns with smoking prevalence in the real world. Specifically, because the male-to-female smoking prevalence ratio in the Lao general population is ~7 (51%:7%) [3], we propose to recruit a sample that includes approximately 15% (n=75) female participants. By doing so, we recognize that we may not have sufficient power to detect a difference in the primary outcome in the female subsample. However, given that little is known about the factors associated with smoking cessation in Laotian females, this proposal will provide valuable information that may inform future research efforts.

### Conclusion

If the proposed project is successful, it has the potential to transform health care services for tobacco treatment throughout Lao PDR and, ultimately, to significantly reduce tobacco-induced morbidity and mortality in the country. The AT’s potential for widespread adoption and sustainability is enhanced by the direct involvement of Lao governmental stakeholders at multiple national institutes. Furthermore, the US-Lao collaborative work and capacity-building activities in this project will contribute to creating a knowledge base for mHealth research applications and advancing mHealth research capacity in Lao PDR.

## Supplementary material

10.2196/89916 Checklist 1 CONSORT-eHEALTH checklist (V 1.6.1).

10.2196/89916 Peer Review Report 1 Peer review report from ZRG1 IMST-K (55) - Center for Scientific Review Special Emphasis Panel PAR-19-376: Mobile Health: Technology and Outcomes in Low and Middle Income Countries (National Institutes of Health, USA).

## Abbreviations

AT: automated treatment
CH: Champasak Hospital
CO: carbon monoxide
CONSORT: Consolidated Standards of Reporting Trials
GLMM: generalized linear mixed model
IRB: institutional review board
Lao PDR: Lao People's Democratic Republic
LMIC: low- and middle-income country
mHealth: mobile health
MOH: Ministry of Health
NCI: National Cancer Institute
NRT: nicotine replacement therapy
NTCC: National Tobacco Control Committee
PBM: phase-based model
PI: principal investigator
RCT: randomized controlled trial
RDSU: respiratory disease screening unit
REDCap: Research Electronic Data Capture
SC: standard care
SH: Setthathirath Hospital
SPIRIT: Standard Protocol Items: Recommendations for Interventional Trials
WHO: World Health Organization
