# Supplementary material for: Mobile Health Technology for Personalized Tobacco Cessation Support in Laos (Project Support Laos): Protocol for a Randomized Controlled Trial
Source: JMIR Res Protoc. 2026 May 21;15:e89916. doi: 10.2196/89916 (PMC13193577; doi:10.2196/89916)
Supplement: Peer Review Report 1 [file resprot-v15-e89916-s002.pdf]

SUMMARY STATEMENT

(Privileged Communication)

PROGRAM CONTACT:
Vidya Vedham
240-276-7272
vidya.vedham@nih.gov

Release Date: 02/29/2020
Revised Date:

Application Number: 1 R21 CA253600-01

Principal Investigator

BUI, THANH C

Applicant Organization: UNIVERSITY OF OKLAHOMA HLTH SCIENCES CTR

Review Group: ZRG1 IMST-K (55)
Center for Scientific Review Special Emphasis Panel
PAR-19-376:Mobile Health: Technology and Outcomes in Low and Middle Income Countries

Meeting Date: 02/19/2020
Council: MAY 2020
Requested Start: 07/01/2020

RFA/PA: PAR19-376
PCC: AEGH
Dual IC(s): TW

Project Title: Mobile Health Technology for Personalized Tobacco Cessation Support in Laos

SRG Action: Impact Score:28 Percentile:11 #
Next Steps: Visit https://grants.nih.gov/grants/next_steps.htm
Human Subjects: 30-Human subjects involved - Certified, no SRG concerns
Animal Subjects: 10-No live vertebrate animals involved for competing appl.
Gender: 1A-Both genders, scientifically acceptable
Minority: 5A-Only foreign subjects, scientifically acceptable
Age: 3A-No children included, scientifically acceptable

Project Year	Direct Costs Requested	Estimated Total Cost
1	124,902	165,965
2	123,897	164,629
3	198,794	264,149
4	196,794	261,492
5	199,476	265,055
TOTAL	843,863	1,121,290

1R21CA253600-01 BUI, THANH

RESUME AND SUMMARY OF DISCUSSION: This application proposes to adapt a mobile health based intervention to promote smoking cessation in Lao People's Democratic Republic (Lao PDR) using an already developed platform called Insight. If successful, this intervention will curb the high rate of smokers in Lao PDR. During the discussion, the review panel noted many strengths including, an important problem tackled, the first program in Lao PDR, a good team with a similar pilot program in Cambodia, a rigorous approach with a good model, and an excellent capacity building with local engagement. However, the reviewers were concerned that the innovation was incremental, and the intervention support was unclear. They found the local pharmaceutical resources unclear. Finally, there was much discussion about the transition milestones between R21 and R33. Some reviewers found defined milestones while others did not see a specific cut-off quantified. At the end of the discussion, the review panel concluded that this application would exert a high impact on the field of smoking cessation in low and middle income countries.

DESCRIPTION (provided by applicant): Tobacco use remains the leading cause of preventable morbidity and mortality worldwide. Although tobacco use has declined in developed nations in recent decades, smoking prevalence remains strikingly high in many low- and middle-income countries (LMICs). In Lao People's Democratic Republic (Lao PDR), 51% of adult men and 7% of adult women smoke tobacco. The development and evaluation of sustainable tobacco cessation interventions suitable for widespread adoption in nations such as Lao PDR are pressing public health needs. To address this need, we propose a project that adapts our theoretically and empirically based mobile health (mHealth) technology to help people quit smoking cigarettes in Lao PDR. This mHealth approach includes a fully automated, interactive, personalized, smartphone- delivered intervention for behavioral treatment, delivered through our Insight™ platform. mHealth interventions are proliferating in developed countries, but efforts to utilize similar approaches in Lao PDR are extremely limited. The World Health Organization acknowledges that using mHealth for health promotion is cost-effective, scalable, and sustainable, including for the least-developed countries. Additionally, text-messaging interventions for smoking cessation have been shown to be effective, cost-effective, and affordable for tobacco control globally. We have already developed the mHealth platform and intervention approach proposed for this study and pilot tested this technology with a group of Cambodian smokers. Preliminary results demonstrated that our mHealth technology is efficacious, highly feasible, appropriate, and potentially scalable for LMIC settings. This proposed project for Lao PDR includes 2 main phases. In the R21 Phase, we will use formative research methods to adapt our intervention content to the sociocultural context, language, and communication styles of Laotians. In the subsequent R33 Phase, we will conduct a randomized controlled trial (RCT) to evaluate the efficacy of our mHealth intervention and technology. Adult smokers of both sexes will be recruited through 2 large hospitals: Setthathirath Hospital in Vientiane and Champasak Hospital in Champasak Province. Participants (n=500) will be randomized to 1 of 2 treatment groups: Standard Care (SC; n=250) or Automated Treatment (AT; n=250). SC consists of brief advice to quit smoking delivered by research staff, self-help written materials, and a 2-week supply of NRT (transdermal patches). AT consists of all SC components plus a fully automated smartphone-based treatment program that involves interactive and personalized proactive messages, images, or videos. The primary health outcome of the trial is biochemically confirmed self- reported 7-day point prevalence abstinence 12 months post study enrollment. The project also aims to advance mHealth research capacity in Lao PDR and sustain the US-Lao PDR research network. The project has the potential to transform healthcare services for tobacco cessation treatment throughout the country and, ultimately, to significantly reduce tobacco-induced morbidity and mortality.

PUBLIC HEALTH RELEVANCE: This project is a collaborative initiative between US researchers and Lao public health officials to determine the efficacy of a novel mHealth intervention for smoking cessation among Lao patients. The proposed research is relevant to public health because it is expected to advance mHealth applications in tobacco-cessation treatment in low- and middle-income settings and subsequently reduce tobacco-related morbidities and mortality. The mHealth intervention is also affordable and scalable and thus is appropriate for widespread adoption in low- and middle-income countries.

CRITIQUE 1

Significance: 2
Investigator(s): 3
Innovation: 2
Approach: 3
Environment: 2

Overall Impact: This application proposes to conduct a two phase study of R21 and R33 to adapt a mHealth based intervention to promote smoking cessation in Lao PDR. Their proposed mHealth approach includes a fully automated, interactive, personalized, smartphone delivered intervention for behavioral treatment, delivered through an “Insight™ platform”. The R21 Phase will use formative research methods to adapt the proposed intervention content to the sociocultural context, language, and communication styles of Laotians. In the subsequent R33 Phase, the applicants proposes to conduct a randomized controlled trial (RCT) to evaluate the efficacy of the mHealth intervention and technology. The study is somehow innovative in the context of Lao PDR, as this will use a fully automated smartphone based treatment program to support smoking cessation. This is a multidisciplinary research team who have worked in similar programs in the past; inclusion of several Lao based investigators ensures the local engagement in the proposed work. There are potential for mHealth capacity building in Lao PDR through the implementation of this study. The milestones for R21 and transition from R21 to R33 is appropriate and seems feasible. Although the project has good potential to be integrated within the Lao healthcare setting by collaborating with the Ministry of Health (MoH), the commitment of the MoH sometimes does not reflect into actions and requires additional advocacy work.

1. Significance:

Strengths

- Tobacco use in Lao PDR is a significant health problem (51% of men and 7% of women smokes cigarette) and innovative approaches to address tobacco use reduction and cessation is needed.
- There are lack of theory based tobacco cessation services in the country, and this project could address this deficit by integrating the intervention within the healthcare setting.
- mHealth based intervention has shown effectiveness in developed countries to support cessation and this may offer hope for the LMICs (i.e Lao PDR)

Weaknesses

- The current situation of tobacco cessation services in the country is not well described. What is the overall willingness to quit among the population?
- What types of pharmacological products to support smoking cessation is available locally and how they are financially covered?

2. Investigator(s):

Strengths

- Dr. Bui is a Research Assistant Professor at the University of Oklahoma Health Sciences Center and will be the PI for the proposed study. The PI has assembled a multidisciplinary team of researchers to support the proposed work in Lao.
- The team comprised of experienced researchers with previous collaborative experience in similar studies.
- The team member's strength are complementary and cover diverse aspects of the proposed study.
- The PI has conducted research in the region but not in Lao PDR.
- The coinvestigators from Lao are qualified to support the local activities and support future expansion.

Weaknesses

- As the PI is a junior faculty, supports from other team members would be needed for the appropriate implementation of the study.
- Not sure if the PI (Dr. Bui) can communicate in Lao language as communication in English is a problem in the country. Always relying on bi-lingual coinvestigators may not be effective.
- None of the team members seems to have worked with the team in Lao PDR; so this will be a new collaboration.

3. Innovation:

Strengths

- The use of mHealth (use of the US-based Insight flatform) to support smoking cessation is innovative for Lao PDR, as the concept is not totally new.
- The use of phase based model model (PBM) to adapt/develop the intervention is innovative.

Weaknesses

- The Insight platform was developed in the US; and, hence the need for cultural adaptation will be critical.

4. Approach:

Strengths

- The approaches described for the R21 phase and R33 phase is appropriate and feasible.
- Overall design of formative study to develop and adapt the intervention is logical.
- The cultural tailoring of the intervention following the 5 stages as described by Barrera et al., is appropriate.

- In R21 phase, separate group discussion for men and women is appropriate given the male/female power dynamics in Asian culture.
- The capacity building component is well described, which may contribute to the mHealth research capacity development in Lao PDR.
- Data Management and Statistical Analysis is appropriate.

Weaknesses

- The diversity of the patient population attending these two hospitals (though in 2 separtae regions) may not be representative of the total adult smokers in the country. However, these settings may work as a starting point to expand the work through other medium/small type hospital settings.
- In R21 phase, use of purposive sampling to gather data from diverse population (n=30) is good. However, so many criteria are mentioned (i.e. age, residence, disease type, cessation phase) without specifying the number of subjects in each category to be included.
- The types of pharmacological products to support smoking cessation available locally is not described. How the current offer of 2 week NRT will be sustained within the healthcare setting in the future?

5. Environment:

Strengths

- Strong research environment in both the US and Lao DPR partner sites.
- The primary institution, University of Oklahoma Health Science Center (OUHSC), has the adequate facilities to carry out the proposed work. Other participating institutions has adequate resources to support the proposed research.

Weaknesses

- Limited resources are available in the participating agencies in Lao PDR, but should be adequate to support the current study.

- The collaboration between the US and the Lao partners is new. Therefore, the potential for the continuation of the mHealth research, upon completion of the current proposal, is uncertain.

Milestones:

Strengths

- Transition milestones are well described in quantifiable terms.
- The milestones, as described for R21 phase of the study, seems to me achievable; which will lead transition to the R33 phase of the study.

Weaknesses

- Evaluation of their readiness to start the R33 phase is not clear. Will they have any advisory committee or other group meeting to assess the progress with the R21 phase before starting R33?

Study Timeline:

Strengths

- Study timeline described for both the R21 phase and R33 phase is appropriate and seems to me feasible.
- Several planned activities within the proposed timeline, if achieved, will enhance the expansion and continuation of the proposed work.

Weaknesses

- Potential problems and alternative strategies are not described.

Protections for Human Subjects: Acceptable Risks and/or Adequate Protections

- Human subjects risk details and mitigation plan is well described.

Data and Safety Monitoring Plan (Applicable for Clinical Trials Only):

Acceptable

- o DSMB will be formed and include members from both US and Lao institutions.

Inclusion Plans: Applicable Only for Human Subjects research and not IRB Exemption #4.

- Sex/Gender: Distribution justified scientifically
- Race/Ethnicity: Distribution justified scientifically
- For NIH-Defined Phase III trials, Plans for valid design and analysis: Not applicable
- Inclusion/Exclusion Based on Age: Distribution justified scientifically
- Those aged 18 and above (legal age to consent) will be included

Vertebrate Animals:

Not Applicable (No Vertebrate Animals)

Biohazards:

Not Applicable (No Biohazards)

Applications from Foreign Organizations

Not Applicable (No Foreign Organizations)

Select Agents:

Not Applicable (No Select Agents)

Resource Sharing Plans:

Acceptable

Authentication of Key Biological and/or Chemical Resources: Not Applicable (No Relevant Resources)

Budget and Period of Support:

Recommend as Requested

Recommended budget modifications or possible overlap identified:

CRITIQUE 2

Significance: 1

Investigator(s): 2

Innovation: 5

Approach: 2

Environment: 2

Overall Impact: Strong proposal. Only limitation is that it is essentially repeating just completed work in Cambodia and therefore lacks innovation.

1. Significance:

Strengths

- Smoking is one of highest in the world among men.

no national tobacco treatment programs are available

The MOH's receptiveness and support of mHealth solutions in other domains (e.g. vaccine reporting) *increase the potential for sustainability and widespread adoption of proposed mHealth intervention approach.*

Weaknesses

- none

2. Investigator(s):

Strengths

- Strong track record of doing similar work and being successful in Cambodia.
- Team has worked together in Laos at least—Laos US parts-- since 2017

Weaknesses

- none

3. Innovation:

Strengths

- Insight Platform is innovative—combines automated and staff support. Through one-click buttons in the InsightTM app, participants can call or message project staff if they have questions or need assistance
- Advantage over SMS is that Our InsightTM app will automatically deliver all interventions components ***with or without an active connection***
- Our project will provide a strong foundation for ***future continued applications of mHealth intervention research*** in Lao PDR.
- Only mhealth program in Laos

Weaknesses

- Seems like same work as Cambodia. Unclear that switching countries makes it innovative. This is the primary change.
- smartphones may currently exclude some very low-income smokers

4. Approach:

Strengths

- R21 phase involves many iterations and methodologically sound for developing the proposed product culminating in a 3-month pilot efficacy trial with 50 patients. Half of participants are female.
- will provide smartphones for those who do not have it.
- 2 arm RCT is appropriate.

Weaknesses

- Can’t oversample females for main trial because of short time frame.
- Approach is adequate.

5. Environment:

Strengths

- Team has pilot work

Weaknesses

- New collaborations but prior pilot work.

Milestones:

Strengths

- Well specified in table 1 and include acceptability, feasibility and preliminary efficacy.

Weaknesses

- none

Study Timeline:

Strengths

- appropriate

Weaknesses

- none

Protections for Human Subjects:

Acceptable Risks and/or Adequate Protections

Data and Safety Monitoring Plan (Applicable for Clinical Trials Only):

Not Applicable (No Clinical Trials)

Inclusion Plans: Applicable Only for Human Subjects research and not IRB Exemption #4.

- Sex/Gender: Distribution justified scientifically
- Race/Ethnicity: Distribution justified scientifically
- For NIH-Defined Phase III trials, Plans for valid design and analysis: Not applicable
- Inclusion/Exclusion Based on Age: Distribution justified scientifically

Vertebrate Animals:

Not Applicable (No Vertebrate Animals)

Biohazards:

Not Applicable (No Biohazards)

Applications from Foreign Organizations

Not Applicable (No Foreign Organizations)

Select Agents:

Not Applicable (No Select Agents)

Resource Sharing Plans:

Not Applicable (No Relevant Resources)

Authentication of Key Biological and/or Chemical Resources: Not Applicable (No Relevant Resources)

Budget and Period of Support:

Recommend as Requested

Recommended budget modifications or possible overlap identified:

CRITIQUE 3

Significance: 1
Investigator(s): 1
Innovation: 3
Approach: 2
Environment: 1

Overall Impact: The proposal describes using an mHealth tool based on an existing platform called Insight to assist patients to quit smoking tobacco in Lao PDR, which has a very rate of smokers. Insight is a “fully automated, interactive, personalized, smartphone-delivered intervention for behavioral treatment.” This tool has shown promising pilot results in Cambodia even without the use of NRT.

During the R21 phase, the team proposes to adapt the intervention for Lao PDR. During the R33 phase, an RCT with 500 smokers will be conducted. Primary outcome is biochemically confirmed self-reported 7-day point prevalence abstinence at 12 months. The team has a strong collaboration, with involvement of the MOH, enhancing potential impact if the study is successful.

The proposal describes a clear and important scientific question and employs appropriate design to address it. The team has the requisite skills and experience and if successful, the project has the potential to make a substantial impact, both in Laos and elsewhere. Few minor details in the research approach slightly diminish some enthusiasm but the overall potential impact of the project is moderate to high.

1. Significance:

Strengths

- Tobacco use a major global health problem.
- This is particularly worse in LMICs, given the combination of weaker anti-tobacco policies and lack of treatments.
- 51% of men in Lao PDR are smokers.
- Although policy changes have been made in Lao, treatments are not available, further increasing the importance of this study.
- Mobile cellular subscription rates in Lao are lower than the regional average, potentially limiting the overall impact. However, ownership rates are trending up, providing optimism that by the time this study is completed, national scale-up may be feasible.

Weaknesses

- None

2. Investigator(s):

Strengths

- There is a multidisciplinary, international team with expertise in the relevant domains of mhealth, health promotion, tobacco cessation, mixed-methods, global health, and biostatistics.
- There is history of collaboration between the PI and the Lao-based team members.
- The team has conducted a successful pilot in Cambodia, increasing the likelihood of success in Lao.

Weaknesses

- None

3. Innovation:

Strengths

- Use of automated tools to encourage smoking cessation using personalized messages.
- Use of a versatile, existing platform Insight, which has been used for 40 health studies.
- App can deliver key messages without the need for an active cellular signal.
- Framework-driven cultural adaptation process.
- Phase-based model to guide intervention components to advance the patient along the stages of behavior change.

Weaknesses

- There have been several studies of smoking cessation using automated messages in other parts of the world.

4. Approach:

Strengths

- Detailed description of intervention adaptation in the R21 stage
- WHO-recommended steps in appropriate translation of materials.
- Incorporating male and female smokers, and healthcare workers in the formative stages.
- The proposal outlines several key considerations on their decision-making process that led to the proposed study design (NRT use, smartphone loans and concerns regarding oversampling female smokers).
- The RCT design is positioned to provide well-powered conclusions.
- Detailed plan to ensure adequate enrolment is achieved. High volume facilities with national programs increase likelihood of achieving recruitment targets.
- Paradata are collected by the app's activity log.
- Use of CO monitor to confirm self-reports.
- A plan for missing data and use of intention to treat analysis.
- Statistical analysis is well-described.
- Clearly detailed plans to build capacity with activities that can be assessed for this important aspect of the funding mechanism (who will learn which research skills by when and how will this be assessed).

Weaknesses

- A 3-month efficacy trial with 50 patients may be difficult to achieve, given that it will be a new app that may generate errors. Given that this intervention has already showed promising pilot data, albeit in a different country, the team may consider reducing the N for the efficacy trial to increase feasibility and facilitate timely transition to the R33 phase.
- Focus on patients who are already motivated to quit slightly decreases the enthusiasm for impact while recognizing that enhancing motivation for precontemplative or contemplative stage patients is a substantial challenge that is unlikely to be addressed by mHealth tools and likely requires in-person motivational interviewing.
- Given the inclusion criteria of ≥1 cigarette/day, there is risk that this will sample light smokers and not heavier smokers. The distribution of light vs. heavy smokers is unclear from the prevalence data provided but the researchers may consider ensuring accurate representation of both categories, given the differential challenges in these two groups, since they will already be assessing for nicotine dependence using the heaviness of smoking index.
- The use of patch only as NRT is not adequately described or justified. Given the inclusion criteria of ≥1 cigarette/day, even the lowest dose patch may be more than necessary for those who smoke fewer than 7 cigarettes a day. In addition, patches require a stepped approach that often lasts several weeks but the proposal notes providing only a 2-week supply. It is not clear why gums or other NRT products are not used for cases where patch may not be the best option.
- In addition, as NRT will be given for 2 weeks, it is not clear what happens if patients request/require NRT for longer than that.

5. Environment:

Strengths

- The facilities section provides ample evidence of support for the proposed research in the research team's institutions.

Weaknesses

- None

Milestones:

Strengths

- Detailed timeline with milestones is available. Specific deliverables are listed, allowing tracking of progress.

Weaknesses

- None

Study Timeline: (Specific to applications designated clinical trial on the electronic cover sheet)

Strengths

- Detailed timeline with milestones is available. Specific deliverables are listed, allowing tracking of progress.

Weaknesses

- None

Protections for Human Subjects: Acceptable Risks and/or Adequate Protections

Data and Safety Monitoring Plan (Applicable for Clinical Trials Only):

Acceptable

Inclusion Plans: Applicable Only for Human Subjects research and not IRB Exemption #4.

- Sex/Gender: Distribution justified scientifically
- Race/Ethnicity: Distribution justified scientifically
- For NIH-Defined Phase III trials, Plans for valid design and analysis:
- Inclusion/Exclusion Based on Age: Distribution justified scientifically

Applications from Foreign Organizations

Justified

Budget and Period of Support:

Recommend as Requested

Recommended budget modifications or possible overlap identified:

THE FOLLOWING SECTIONS WERE PREPARED BY THE SCIENTIFIC REVIEW OFFICER TO SUMMARIZE THE OUTCOME OF DISCUSSIONS OF THE REVIEW COMMITTEE, OR REVIEWERS' WRITTEN CRITIQUES, ON THE FOLLOWING ISSUES:

PROTECTION OF HUMAN SUBJECTS: ACCEPTABLE

INCLUSION OF WOMEN PLAN: ACCEPTABLE

INCLUSION OF MINORITIES PLAN: ACCEPTABLE

INCLUSION ACROSS THE LIFESPAN PLAN: ACCEPTABLE

COMMITTEE BUDGET RECOMMENDATIONS: The budget was recommended as requested.

Footnotes for 1 R21 CA253600-01; PI Name: BUI, THANH C

Ad hoc or special section application percentiled against "Total CSR" base.

NIH has modified its policy regarding the receipt of resubmissions (amended applications).See Guide Notice NOT-OD-18-197 at https://grants.nih.gov/grants/guide/notice-files/NOT-OD-18-197.html. The impact/priority score is calculated after discussion of an application by averaging the overall scores (1-9) given by all voting reviewers on the committee and

multiplying by 10. The criterion scores are submitted prior to the meeting by the individual reviewers assigned to an application, and are not discussed specifically at the review meeting or calculated into the overall impact score. Some applications also receive a percentile ranking. For details on the review process, see http://grants.nih.gov/grants/peer_review_process.htm#scoring.

MEETING ROSTER

Center for Scientific Review Special Emphasis Panel
CENTER FOR SCIENTIFIC REVIEW
PAR-19-376:Mobile Health: Technology and Outcomes in Low and Middle Income Countries

ZRG1 IMST-K (55)
02/19/2020 - 02/20/2020

Notice of NIH Policy to All Applicants: Meeting rosters are provided for information purposes only. Applicant investigators and institutional officials must not communicate directly with study section members about an application before or after the review. Failure to observe this policy will create a serious breach of integrity in the peer review process, and may lead to actions outlined in NOT-OD-14-073 at https://grants.nih.gov/grants/guide/notice-files/NOT-OD-14-073.html and NOT-OD-15-106 at https://grants.nih.gov/grants/guide/notice-files/NOT-OD-15-106.html, including removal of the application from immediate review.

CHAIRPERSON(S)

SPRUIJT-METZ, DONNA D, PHD
RESEARCH PROFESSOR AND DIRECTOR
USC MHEALTH COLLABORATORY
CENTER FOR ECONOMIC AND SOCIAL RESEARCH
UNIVERSITY OF SOUTHERN CALIFORNIA
LOS ANGELES, CA 90089

MEMBERS

ABDULLAH, ABU SALEH, MD, PHD
RESEARCH ASSOCIATE PROFESSOR
BOSTON MEDICAL CENTER
BOSTON UNIVERSITY SCHOOL OF MEDICINE
BOSTON, MA 02118

ABROMS, LORIEN C, SCD, MA
PROFESSOR
MILKEN INSTITUTE SCHOOL OF PUBLIC HEALTH
DEPARTMENT OF PREVENTION AND COMMUNITY HEALTH
GEORGE WASHINGTON UNIVERSITY
WASHINGTON, DC 20037

ACHARYA, BIBHAV, MD
ASSISTANT PROFESSOR
DEPARTMENT OF PSYCHIATRY
SCHOOL OF MEDICINE
UNIVERSITY OF CALIFORNIA AT SAN FRANCISCO
SAN FRANCISCO, CA 94143-0962

BARCLAY, GILLIAN R., DDS, DPH
ACADEMIC HEALTH SPECIALIST
OFFICE OF THE PRESIDENT
UNIVERSITY OF NEVADA LAS VEGAS
LAS VEGAS, NV 89154

BARNETT, IAN JAMES, PHD
ASSISTANT PROFESSOR
DEPARTMENT OF BIOSTATISTICS AND EPIDEMIOLOGY
PERELMAN SCHOOL OF MEDICINE
UNIVERSITY OF PENNSYLVANIA
PHILADELPHIA, PA 19104

BENNETT, IAN MOORE, MD, PHD
PROFESSOR
DEPARTMENTS OF FAMILY MEDICINE AND
PSYCHIATRY AND BEHAVIORAL SCIENCES
AND THE DEPARTMENT OF GLOBAL HEALTH
UNIVERSITY OF WASHINGTON
SEATTLE, WA 98195

BOIVIN, MICHAEL JOSEPH, PHD
PROFESSOR
DEPARTMENT OF PSYCHIATRY AND
NEUROLOGY/OPHTHALMOLOGY
COLLEGE OF OSTEOPATHIC MEDICINE
MICHIGAN STATE UNIVERSITY
EAST LANSING, MI 48824

BUIS, LORRAINE R, PHD
ASSISTANT PROFESSOR
DEPARTMENT OF FAMILY MEDICINE
UNIVERSITY OF MICHIGAN MEDICAL SCHOOL
ANN ARBOR, MI 48104

BUTLER, LISA MICHELLE, PHD
ASSOCIATE RESEARCH PROFESSOR
INSTITUTE FOR COLLABORATION ON HEALTH,
INTERVENTION, AND POLICY
UNIVERSITY OF CONNECTICUT
STORRS, CT 06269

CAPLAN, SUSAN, PHD
PROFESSOR AND CHAIR
SCHOOL OF NURSING
COLLEGE OF HEALTH AND HUMAN SCIENCES
NORTHERN ILLINOIS UNIVERSITY
DEKALB, IL 60115

CHAWLA, NITESH, PHD
PROFESSOR
DIRECTOR, THE INTERDISCIPLINARY CENTER FOR
NETWORK SCIENCE AND APPLICATIONS
DEPARTMENT OF COMPUTER SCIENCE AND ENGINEERING
UNIVERSITY OF NOTRE DAME
NOTRE DAME, IN 46556

COHEN, JESSICA LEE, PHD
ASSOCIATE PROFESSOR
DEPARTMENT OF GLOBAL HEALTH AND POPULATION
HARVARD T. H. CHAN SCHOOL OF PUBLIC HEALTH
BOSTON, MA 02115

DE ERAUSQUIN, GABRIEL ALEJANDRO, MD, PHD
PROFESSOR AND FOUNDING CHAIR
DEPARTMENT OF PSYCHIATRY AND NEUROLOGY
SCHOOL OF MEDICINE
UNIVERSITY OF TEXAS RIO GRANDE VALLEY
BROWNSVILLE, TX 78520

EHRHARDT, STEPHAN, MPH, MD
ASSOCIATE PROFESSOR
DEPARTMENT OF EPIDEMIOLOGY
JOHNS HOPKINS SCHOOL OF PUBLIC HEALTH
BALTIMORE, MD 21205

GANCE-CLEVELAND, BONNIE, PHD
LORETTA FORD PROFESSOR
COLLEGE OF NURSING
ANSCHUTZ MEDICAL CAMPUS
UNIVERSITY OF COLORADO
AURORA, CO 80045

GILL, CHRISTOPHER J, MD
ASSOCIATE PROFESSOR
DEPARTMENT OF GLOBAL HEALTH
SCHOOL OF PUBLIC HEALTH
BOSTON UNIVERSITY
BOSTON, MA 02118

IYENGAR, MADURAI SRIRAM, PHD
ASSOCIATE PROFESSOR
DEPARTMENT OF MEDICINE
COLLEGE OF MEDICINE
UNIVERSITY OF ARIZONA
PHOENIX, AZ 85004

JONES, DEBORAH LYNNE, BA, MEDS, PHD
PROFESSOR
DEPARTMENT OF PSYCHIATRY
AND BEHAVIORAL SCIENCES
MILLER SCHOOL OF MEDICINE
UNIVERSITY OF MIAMI
MIAMI, FL 33136

LEMMA, WULETA, PHD
ASSOCIATE PROFESSOR
COLLEGE OF MEDICINE AND HEALTH SCIENCES
WOLLO UNIVERSITY
DESSIE
ETHIOPIA

LIU, LONGJIAN, MD, PHD
ASSOCIATE PROFESSOR
DEPARTMENT OF EPIDEMIOLOGY AND BIOSTATISTICS
DREXEL UNIVERSITY
PHILADELPHIA, PA 19102

MARKHAM, CHRISTINE MARGARET, PHD
PROFESSOR AND INTERIM CHAIR
DEPARTMENT OF HEALTH PROMOTION AND
BEHAVIORAL SCIENCES
SCHOOL OF PUBLIC HEALTH
UNIVERSITY OF TEXAS AT HOUSTON
HOUSTON, TX 77030

NGO, VICTORIA KHANH, PHD
ASSOCIATE PROFESSOR
DEPARTMENT OF COMMUNTY HEALTH AND SOCIAL
SCIENCES
CITY UNIVERSITY OF NEW YORK
NEW YORK CITY, NY 10027

NWOGU, CHUKWUMERE E, MD, PHD
ASSOCIATE PROFESSOR
SURGERY AND ONCOLOGY DEPARTMENTS
OF THORACIC SURGERY AND CANCER PREVENTION/
CONTROL
UNIVERSITY AT BUFFALO
SUNY ROSWELL PARK CANCER INSTITUTE
BUFFALO, NY 14263

ROTHERAM-BORUS, MARY JANE, PHD
PROFESSOR
DEPARTMENT OF PSYCHIATRY
DIRECTOR, GLOBAL CENTER FOR CHILDREN AND FAMILIES
SEMEL INSTITUTE FOR NEUROSCIENCE/HUMAN BEHAVIOR
UNIVERSITY OF CALIFORNIA AT LOS ANGELES
LOS ANGELES, CA 90024

SEKANDI, JULIET NABBUYE, MD, DRPH
GLOBAL HEALTH INSTITUTE
COLLEGE OF PUBLIC HEALTH
UNIVERSITY OF GEORGIA
ATHENS, GA 30602

SOLIMAN, AMR, MD, PHD, MPH
PROFESSOR
DEPARTMENT OF COMMUNITY HEALTH AND SOCIAL
MEDICINE
CUNY SCHOOL OF MEDICINE
NEW YORK, NY 10031

SPIEGELMAN, DONNA L, SCD
PROFESSOR
DEPARTMENT OF STATISTICS AND DATA SCIENCE
SCHOOL OF PUBLIC HEALTH
YALE UNIVERSITY
NEW HAVEN, CT 06510

THOMAS, JAMES C, PHD
ASSOCIATE PROFESSOR
DEPARTMENT OF EPIDEMIOLOGY
UNIVERSITY OF NORTH CAROLINA AT CHAPEL HILL
CHAPEL HILL, NC 27599-7400

TORIOLA, ADETUNJI T, MD, PHD
ASSOCIATE PROFESSOR
DEPARTMENT OF SURGERY
DIVISION OF PUBLIC HEALTH SERVICES
WASHINGTON UNIVERSITY SCHOOL OF MEDICINE
ST LOUIS, MO 63110

WALANI, SALIMAH R., MPH, MSN, PHD
VICE PRESIDENT
GLOBAL HEALTH PROGRAMS
MARCH OF DIMES
SCOTTSDALE, AZ 85258

WERE, MARTIN CHIENG, MD
ASSOCIATE PROFESSOR
DEPARTMENT OF BIOMEDICAL INFORMATICS AND MEDICINE
VANDERBILT INSTITUTE OF GLOBAL HEALTH
VANDERBILT UNIVERSITY MEDICAL CENTER
NASHVILLE, TN 37203

SCIENTIFIC REVIEW OFFICER

BELANGER, MARIE-JOSE, PHD
SCIENTIFIC REVIEW OFFICER
CENTER FOR SCIENTIFIC REVIEW
NATIONAL INSTITUTES OF HEALTH
BETHESDA, MD 20892

RICHON, ALLEN, PHD
SCIENTIFIC REVIEW OFFICER
CENTER FOR SCIENTIFIC REVIEW
NATIONAL INSTITUTES OF HEALTH
BETHESDA, MD 20892

EXTRAMURAL SUPPORT ASSISTANT

AKOMAH, STEPHEN
LEAD EXTRAMURAL SUPPORT ASSISTANT
CENTER FOR SCIENTIFIC REVIEW
NATIONAL INSTITUTES OF HEALTH
BETHESDA, MD 20892

Consultants are required to absent themselves from the room during the review of any application if their presence would constitute or appear to constitute a conflict of interest.
